# Evaluating some local grass pea (*Lathyrus sativus* L.) varieties under warm humid continental climate conditions for agricultural sustainability with a special reference to seed yield and β-ODAP content

**DOI:** 10.3389/fpls.2026.1750659

**Published:** 2026-04-15

**Authors:** Uğur Başaran, Yusuf Sarıtaş

**Affiliations:** 1 Department of Field Crops, Faculty of Agriculture, Yozgat Bozok University, Yozgat, Türkiye; 2 Variety Registration and Seed Certification Center, Republic of Türkiye, Ministry of Agriculture and Forestry, Ankara, Türkiye

**Keywords:** genotypic diversity, grass pea (*Lathyrus sativus L.)*, seed yield, Türkish varieties, β-ODAP, SDG 2:zero hunger

## Abstract

Grass pea, historically underutilized as a forage crop, has recently attracted renewed interest due to its exceptional adaptability and minimal input demands, positioning it as a promising contributor to agricultural sustainability and food supply resilience. However, the presence of β-ODAP, a toxic amino acid, has constrained its wider adoption, highlighting the critical need for developing grass pea varieties with low β-ODAP concentrations. In this study, Türkish grass pea varieties were compared among themselves and with world materials in terms of agronomic and biochemical properties. The grass pea varieties evaluated in this study exhibited substantial morphological and chemical diversity and showed acceptable average seed yield (3.11 t ha^-^¹), protein content (28.52%), and phenotypic performance. Overall, the β-ODAP concentrations (3.99 mg g^-^¹) of Türkish varieties were slightly higher than those reported in previous studies worldwide. The results indicated that more recently registered variety “Duduhanım” stands out among the grass pea varieties with its earliness, low thousand grain weight, high grain yield and harvest index, and has an average value in terms of crude protein and B-ODAP content.

## 1 Introduction

General projections for the agricultural sector indicate that it will be extremely difficult for agricultural production to meet increasing food demand in the future. Climate change, the degradation and depletion of soil and water resources, the rise in diseases and pests, and high production costs threaten agricultural sustainability and raise serious concerns about food supply security. Due to climate change, agricultural areas exposed to short or long-term drought are expected to increase in the current century (Dai, 2013). This expectation has made drought tolerance one of the main targets in plant breeding (Cattivelli et al., 2008). For climate-resilient agriculture, diversifying crop production systems with species and varieties resistant to drought and other environmental stressors is of great importance. And, in this context, underutilized crops having favorable characteristics besides stress tolerance are an important complement to sustainable food and feed production (Massawe et al., 2016).

Grass pea (*Lathyrus sativus* L.), is an annual cool-season legume, is the most cultivated species of the *Lathyrus* genus, which includes about 160 species (Asmussen and Liston, 1998). The archaeobotanical data suggests that the origin of cultivation of grass pea goes back to the middle pre-pottery Neolithic age (8200–7500 BCE) in the Balkan Peninsula (Kislev, 1989) and Euphrates Valley (Ferrio et al., 2012). Both locations are located at the two corners of Türkiye.

Despite not currently a major crop globally or locally, grass pea has exhibited a great potential for global food security. Grass pea is uses as a food, feed, fodder and green manure worldwide (Aletor et al., 1994). It could serve as a valuable alternative crop plant for diversifing cropping systems on marginal lands (Vaz Patto et al., 2006). It has remarkable yield stability in yield compared to other legume crops under mild and severe drought (Silvestre et al., 2014) and flooding conditions (Zhelyazkova et al., 2016). It has low input requirements, high protein and nutritional contents in the seeds and leaves with exceptional tolerance to drought, floods, salinity, insect pests, diseases, high and cold temperature (Yang and Zhang, 2005; Vaz Patto et al., 2006; Shubha et al., 2025). However, several varieties of grass pea are recognized for presence of 0.1 - 2.5% or more (w/w) for a neurotoxin β-N-oxalyl-L-α, β-diamino propionic acid (β-ODAP). This neurotoxin cause paralysis of forelimbs in animals, poultry and human beings if consumed at a level that constitutes one-third of the diet (Dufour, 2011) for 3–4 months (Rao et al., 1964; Crews and Clarke, 2014; Xu et al., 2017; Lambein et al., 2019).

Therefore, there is a strong need to breed safe grass pea varieties with low β-ODAP contents and high seed yield. However, the target of breeding zero β-ODAP contents is tedious and has not been achieved yet (Kumar et al., 2011; Ramya et al., 2022). The presence of this compound in the plant species has resulted in its failure to achieve the status of a major sustainable agricultural crop globally (Vaz Patto et al., 2006).

In parallel with the world, recently the interest to the grass pea has increased in Turkey. As of 2024, there are a total of 7 registered grass pea varieties in Turkey, two of them were improved for seed and the others for forage or dual purposes. No specific limit of β-ODAP percentage is required/recognized officially for variety regis tration in Türkiye. This is understandable, because there is no known or recorded case of human, animal or bird based lathyrism in Türkiye. Therefore, this study aimed to compare Türkish grass pea varieties (one of was developed for seed and others for dual purposes) primarily among themselves and with world materials in terms of agronomic properties, grain yield and chemical contents including ODAP content and to reveal the developments in grass pea breeding in Türkiye.

## 2 Materials and methods

Five Türkish registered grass pea varieties (Karadag, Iptas, Eren, Gap Mavisi ve Duduhanım) were used in this study. Of these, Duduhanım is a seed type, while the others are dual-purpose varieties. The seeds were obtained from Prof. Dr. Uğur BAŞARAN’s of Yozgat Bozok University, Faculty of Agriculture, Yozgat, Türkiye. The experiment was conducted over two years (2022 and 2023). The experimental trial started with the sowing of the mentioned cultivars on 1 April 2022 and 27 March 2023 during the spring season at Yozgat (39° 70′ 65″N, 34° 83′ 46″E). Field studies were performed at the location with DSb climates, described as a hot, humid continental climate (Anonymous, 2024).

The trials were arranged according to the randomized block design with 4 replications. Each plot consisted of 6 rows, 500 cm long and 25 cm apart, and was seeded at the rate of 80 seeds per m^2^. The varieties were investigated for agronomic traits, seed yield, nutritional contents (protein, ash, starch, and fiber), and β-ODAP contents in both years.

**Figure 1:**
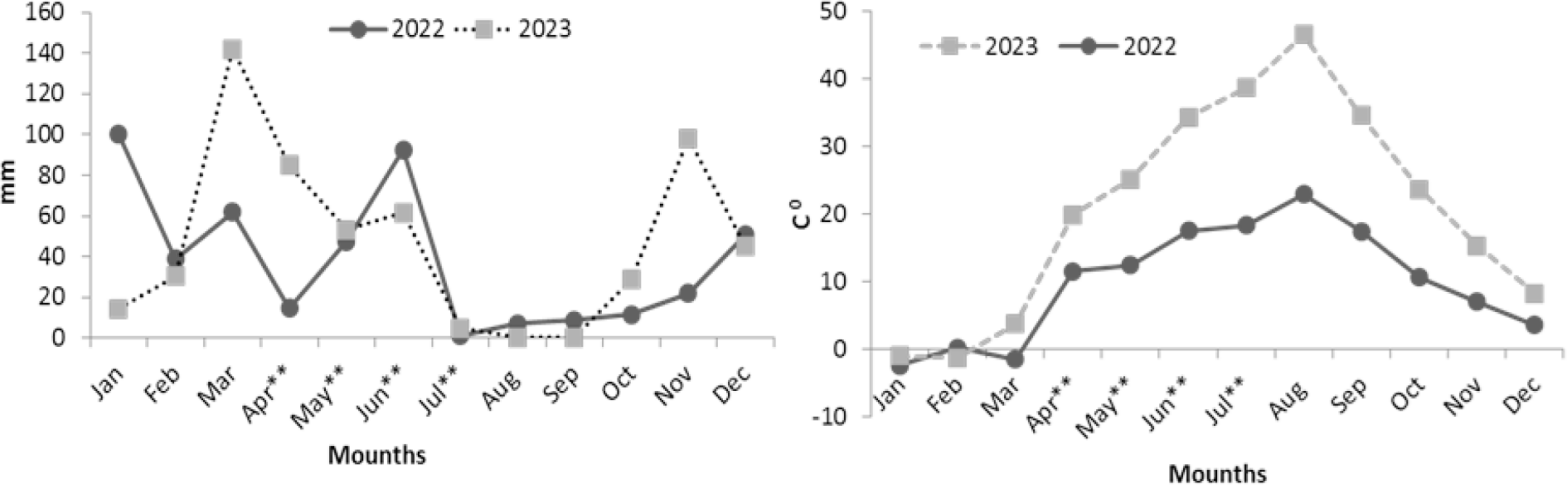
Climatic conditions of experiment area. The graph on the left shows precipitation data, the graph on the right shows temperature data. **months of the vegetation period.

Following planting, 5 kg da^-1^ P_2_O_5_ and 2 kg da^-1^ nitrogen were applied. The experiment was conducted under completely natural climatic conditions and no pesticides or irrigation was used. Hoeing was applied once when the plants were approximately 10 cm tall for weed control. It was followed by manual weeding throughout the experiment as and when required. According to the analysis results of soil samples taken from 30–60 cm depth; the soil structure in the experiment area is clayey, slightly salty, medium calcareous, pH neutral, phosphorus level high and organic matter content medium. Climate data of the experiment area are shown in **Figure 1**. Accordingly, 2023 has been advantageous in terms of rainfall and has also been a warmer year compared to 2022. This situation is also valid during the vegetation period (April-July).

Agronomic characteristics were determined according to VCU (2019). The chemical analysis were performed on dry seed samples ground to pass through a 1 mm sieve. Crude protein was determined using the Kjeldahl procedure following Basaran et al. (2013), with the formula:

Crude protein= [N content ×6.25].

Ash, starch, and fiber analysis were determined by using near-infrared reflectance spectroscopy (NIRS, Foss 6500) with the IC-0904FE software package. This program was initially developed for field pea seeds, and the results have been previously compared with chemical analyses and adapted to grass pea using specific coefficients for each trait. β-ODAP was estimated by O-phthalaldehyde (OPT) assay (Rao, 1978). Reagent was prepared by mixing 100 mg OPT (dissolved in 0.2 ml mercaptoethanol), 1 ml of ethanol (95%) and 100 ml borate buffer (152.7 g potassium tetraborate and 500 ml distilled water). 20 mg of the finely powdered seed sample was placed in a test tube, and 2 ml of distilled water was added. The tubes were kept in boiling water and cooled to room temperature and then centrifuged. 100 μl supernatant + 0.2 ml 3 N KOH were placed in tube and kept in boiling water for 30 minutes. After hydrolysis, 0.7 ml of water and 2 ml OPT regent was added to tube and allowed remain for 15 minutes. Finally, it was measured in spectrophotometer (Perkinelmer Lambda 25) at 425 nm (Basaran et al., 2016).

Statistical analysis: The experimental data for separate and combined years were subjected to analysis of variance and the differences among the means were determined using Duncan’s Multiple Range Test (DMRT) after determining the significance through the F test. In addition, PCA analysis was applied to two-year data.

## 3 Results

**Figure 2:**
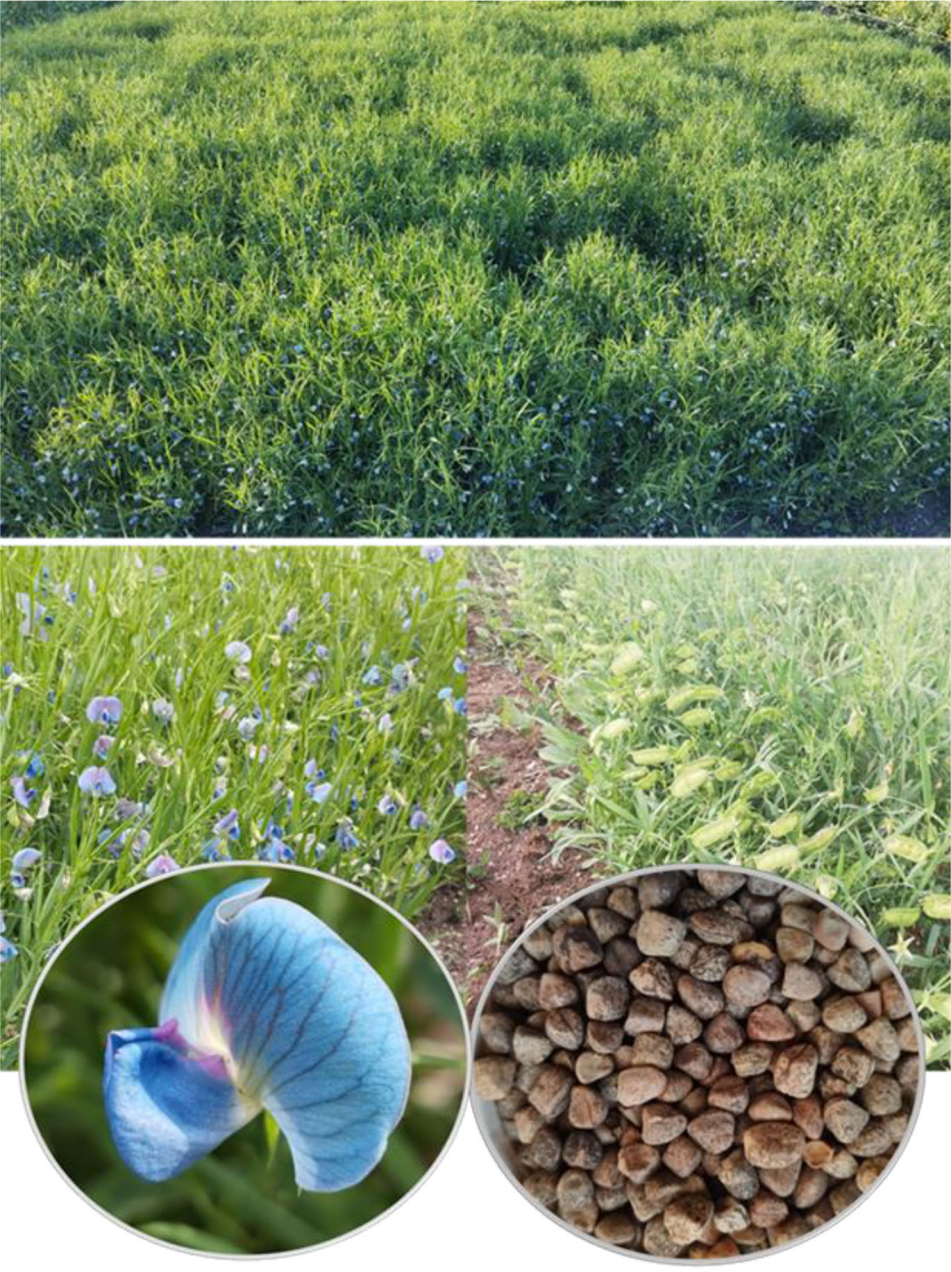
The stages and parts of grass pea (from Duduhanım variety).

The values of the morphological and chemical properties of the examined grass pea varieties determined in 2022 and 2023 are shown in **Tables 1**–**4**. Experimental data were analyzed separately for each year, and the effect of varieties on the evaluated traits was significant, with some exceptions (p<0.01). Furthermore, the average values of these traits over varieties were also statistically different between years. Descriptive images of some developmental periods and parts of the grass pea (specifically the Duduhanım variety) are seen in **Figure 2**.

**Table 1:** The means of biomass (BM), seed (SY) and hay yield (HY) of Türkish grass pea varieties in during 2022 and 2023 years.

Variety	BM (t ha^-1^)		SY (t ha^-1^)		HY (t ha^-1^)	
	2022**	2023	2022**	2023	2022**	2023
Duduhanım	6.30 b	12.15	2.77 a	3.62	3.53 b	8.53
Eren	7.01 ab	11.66	2.50 b	3.41	4.52 a	8.25
Gap Mavisi	7.07 ab	12.34	2.82 a	3.62	4.25 a	8.72
Iptas	7.62 a	11.41	2.64 ab	3.39	4.98 a	8.02
Karadag	7.20 a	12.21	2.72 ab	3.57	4.49 a	8.64
Mean**	7.04 B	11.95 A	2.69 B	3.52 A	4.35 B	8.43 A

**p<0.01, means followed by the same letter at the same column are not statistically different (p<0.05).

**Table 2:** The means of harvest index (HI), thousand-seed weight (TSW) and first flowering day (FFD) of Türkish grass pea varieties in during 2022 and 2023 years.

Variety	HI (%)		TSW (g)		FFD (day)	
	2022**	2023	2022**	2023**	2022**	2023**
Duduhanım	44.00 a	29.83	147.58 b	116.75 d	62.00 d	63.50 d
Eren	35.68 cd	29.28	174.82 a	147.28 b	64.50 b	69.00 a
Gap Mavisi	39.95 b	29.30	174.43 a	137.13 c	63.50 c	64.50 c
Iptas	34.70 d	29.70	176.15 a	155.78 a	66.00 a	68.75 a
Karadag	37.85 bc	29.25	176.23 a	153.73 ab	65.25 ab	66.75 b
Mean**	38.44 A	29.47 B	169.84 A	142.13 B	64.25 B	66.50 A

*p<0.05, **p<0.01, means followed by the same letter at the same column are not statistically different (p<0.05).

**Table 3:** The means of β-ODAP, protein and ash content of Türkish grass pea varieties in during 2022 and 2023 years.

Variety	β-ODAP (mg g^-1^)		Protein (%)		Ash (%)	
	2022**	2023**	2022**	2023**	2022	2023*
Duduhanım	5.26 ab	2.82 b	27.94 b	28.15 b	4.71	4.64 b
Eren	5.51 a	2.30 c	28.47 a	29.44 a	4.72	4.70 ab
Gap Mavisi	4.76 b	3.61 a	28.42 a	29.80 a	4.72	4.71 a
Iptas	5.11 ab	3.03 b	28.33 ab	28.55 b	4.68	4.68 ab
Karadag	4.82 b	2.75 b	27.94 b	28.21 b	4.69	4.69 ab
Mean**	5.09 A	2.90 B	28.22 B	28.83 A	4.71	4.69

*p<0.05, **p<0.01, means followed by the same letter at the same column are not statistically different (p<0.05).

**Table 4:** The means of starch, fat and fiber content of Türkish grass pea varieties in during 2022 and 2023 years.

Variety	Starch (%)		Fat (%)		Fiber (%)	
	2022	2023**	2022**	2023**	2022	2023**
Duduhanım	50.41	52.33 a	1.32 b	1.61 a	7.88	6.90 b
Eren	49.62	50.32 bc	1.69 a	1.41 b	7.69	7.19 ab
Gap Mavisi	49.73	49.05 c	1.36 b	1.61 a	7.77	7.27 ab
Iptas	50.41	50.53 bc	1.46 b	1.66 a	7.45	7.31 a
Karadag	50.54	51.29 ab	1.35 b	1.54 ab	7.63	7.15 ab
Mean**	50.14 B	50.70 A	1.44 B	1.56 A	7.68 A	7.17 B

**p<0.01, means followed by the same letter at the same column are not statistically different (p<0.05).

All varieties used were evaluated to biomass, seed and hay yield and exhibited significantly (p<0.01) differences 2022 but have similar values in 2023. And these three traits averaged higher in 2023 over the genotypes (**Table 1**). These results indicate that both genotype and agroecology highly effective on growth and productivity of grass pea. The lowest biomass yields of 6.30 t ha^-1^ was recorded in Duduhanım in 2022, and the rest of the varieties were placed in similar groups having values between 7.62 and 7.01 t ha^-1^. The biomass yield of all varieties in 2023 was statistically similar with a range of 11.41 to 12.34 t ha^-1^.

The average seed yield among varieties was recorded as 2.69 and 3.52 t ha^-1^ in 2022 and 2023, respectively. When the years are examined separately, the seed yield of the varieties were statistically different (p<0.01) in 2022 and similar in 2023. In 2002, Duduhanım variety was in the highest group in terms of seed yield with 2.77 t ha^-1^. And also, in 2023, Duduhanım and Gap Mavisi were relatively above the other varieties with a yield of 3.62 t ha^-1^. The average hay yield over the varieties was higher in 2023 (8.43 t ha^-1^) than in 2022 (4.35 t ha^-1^). In 2022, significant differences in hay yield were observed among varieties, with Duduhanım having the lowest yield (3.53 t ha^-1^). In 2023, increases of approximately 50% in grass yield were observed in all varieties, but the difference between them was not significant (**Table 1**).

The means of harvest index, thousand-seed weight and first flowering day of grass pea varieties examined in 2022 and 2023 were given in **Table 2**. The average harvest index was 38.44% in 2022 and 29.47 in 2023. Duduhanım stood out with its high harvest index in both years (44.00 and 29.83%, respectively), but in 2023, all varieties were similar in terms of harvest index. The average thousand-grain weight, which was 169.84 g in 2022, was recorded significantly lower (142.13 g) in 2023. The difference between the varieties was significant in both years, and Duduhanım ranked behind all varieties with 147.58 g and 116.75 g, respectively. The first flowering day was noted to compare the varieties regarding for maturity period and the earliest flowering variety in both years was Duduhanım with 62.00 days in 2022 and 63.50 days in 2023. The latest maturing variety was Iptas (66.00 days) in 2022 and Eren (69.00 days) in 2023 (**Table 2**).

The β-ODAP content of grass pea seeds was significantly affected by year and variety (p<0.01); while the average value among varieties was 5.09 mg g^-1^ in the first year of the study, it decreased by approximately 40% to 2.90 mg g^-1^ in the second year (**Table 3**). The β-ODAP content of the varieties ranged from 4.76 to 5.51 mg g^-1^ in 2022; Gap Mavisi and Karadag had the lowest, and Duduhanım had the highest with 5.26 mg g^-1^. In 2023, the β-ODAP content of the varieties decreased, ranging from 2.30 to 3.61 mg g^-1^, with the highest and lowest β-ODAP values ​​recorded in Gap Mavisi and Eren varieties, respectively, while Duduhanım had moderate β-ODAP content (2.82 mg g^-1^).

Among the examined grass pea varieties, the average protein content was measured as 28.22% in 2022 and increased to 28.83% in 2023 (**Table 3**). Eren and Gap Mavisi varieties had the highest protein content in both years. Duduhanım was located in the lowest group in both years with 27.94%, 28.15% content, respectively. Average ash content across varieties was similar in both years (**Table 3**). The ash percentage of all varieties remained statistically similar with a numerical range of 4.68-4.72% in 2022. Variety effect was significant in 2023, with the highest ash content (4.71%) found in the Gap Mavisi, while all other varieties were in the lowest group. Compared to other varieties, Duduhanım had average or slightly below average ash content in both years, with 4.71% and 4.64%, respectively.

Starch, fat and fiber contents of grass pea seeds were also examined and it was seen that all of them were affected significantly (p<0.01) from year to year (**Table 4**). The starch percentage changed among varieties in the range of 49.62% (Eren) - 50.54% (Karadag) in 2022 with no significant statistical differences. However, a significant (p<0.01) variation was detected in the starch contents of the varieties in 2023, ranging from 49.05% (Duduhanım) to 52.33% (Gap Mavisi). The fat percentage showed a variance (p<0.01) among varieties in the range of 1.32-1.69% in 2022 (**Table 4**). Eren had the highest fat content, and all other varieties were statistically similar. The variation in fat percentage changed in 2023 in the range of 1.41-1.66% (p<0.01). The maximum fat percentages was recorded in Iptas and, except Eren, other varieties were statistically similar to Iptas in fat percentages. The fiber percentage had a nonsignificant variance among varieties in 2022 and changed between 7.45-7.88% (**Table 4**). The fiber percentage decreased in 2023 and was remarkably different among varieties and had a range of 6.90-7.31%. The maximum and minimum fiber percentages were noted in Iptas and Duduhanım in the same order in 2023, and the rest of the varieties were in the same group as Duduhanım.

**Figure 3:**
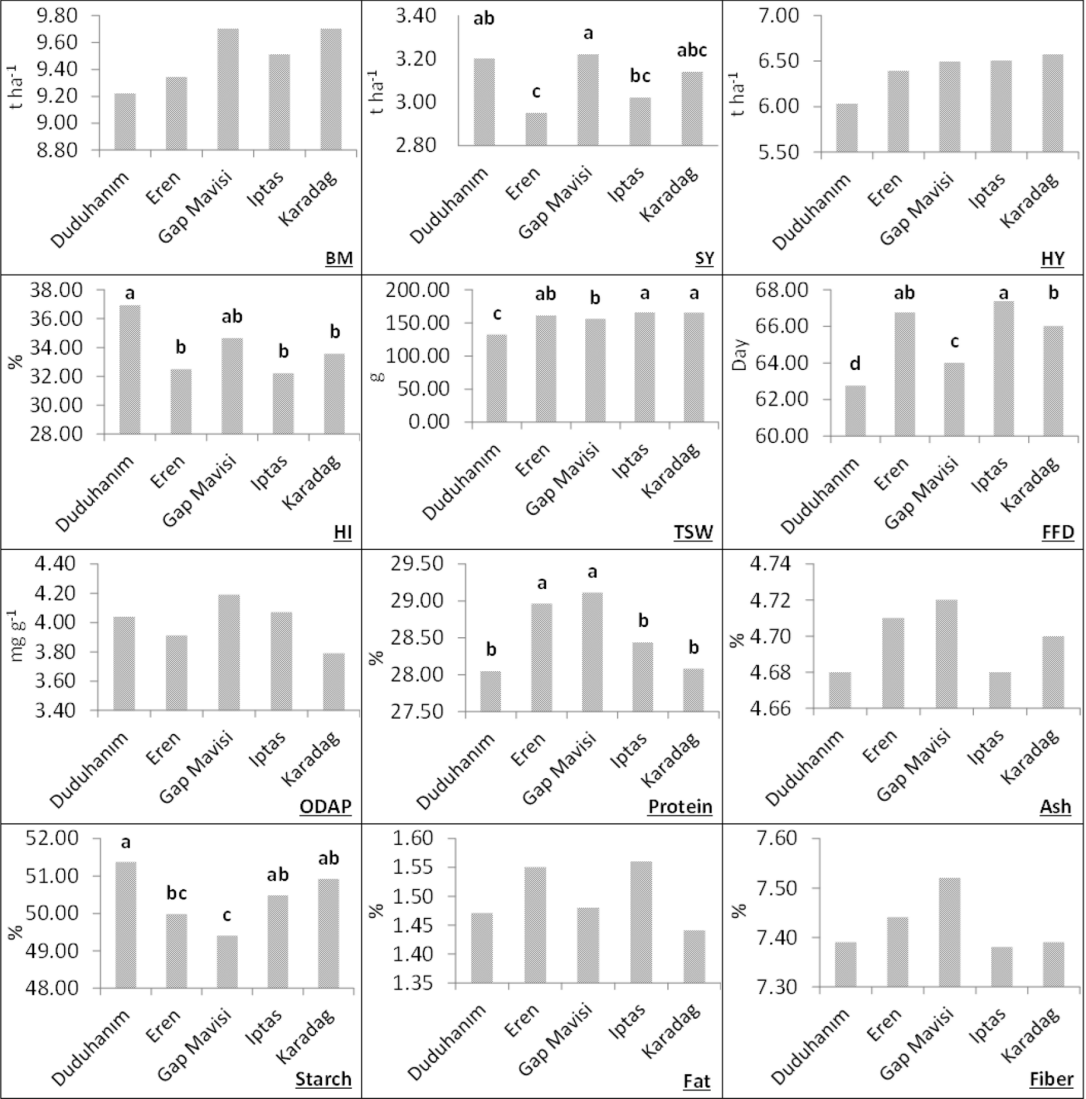
Mean values of the investigated traits of Türkish grass pea varieties in combined years (2022-2023). Means followed by the same letter at the same column are not statistically different (p<0.05), BM, biomass yield; SY, seed yield; HY, hay yield; HI, harvest index; TSW, thousand-seed weight; FFD, first flowering day.

When two years of data are combined, no significant difference was detected among grass pea varieties in terms of biomass yield, β-ODAP, ash, oil, and fiber, however, a significant change (p<0.01) was detected in terms of seed yield, harvest index, thousand-seed weight, fist flowering day thousand, protein and starch (**Figure 3**). These results revealed that the varieties differed in the important traits like seed yield and protein content, but their β-ODAP contents remained similar. Duduhanım and GAP Mavisi are the latest registered varieties. The highest harvest index and seed yield were determined in these varieties based on two-year average data. Two-year averages also indicate that Eren and Gap Mavisi outperform other varieties in terms of protein content with values ​​of 28.96% and 29.11%. Starch content was also significantly different among varieties and the lowest starch content of 49.39% was noted in Gap Mavisi (**Figure 3**).

**Figure 4:**
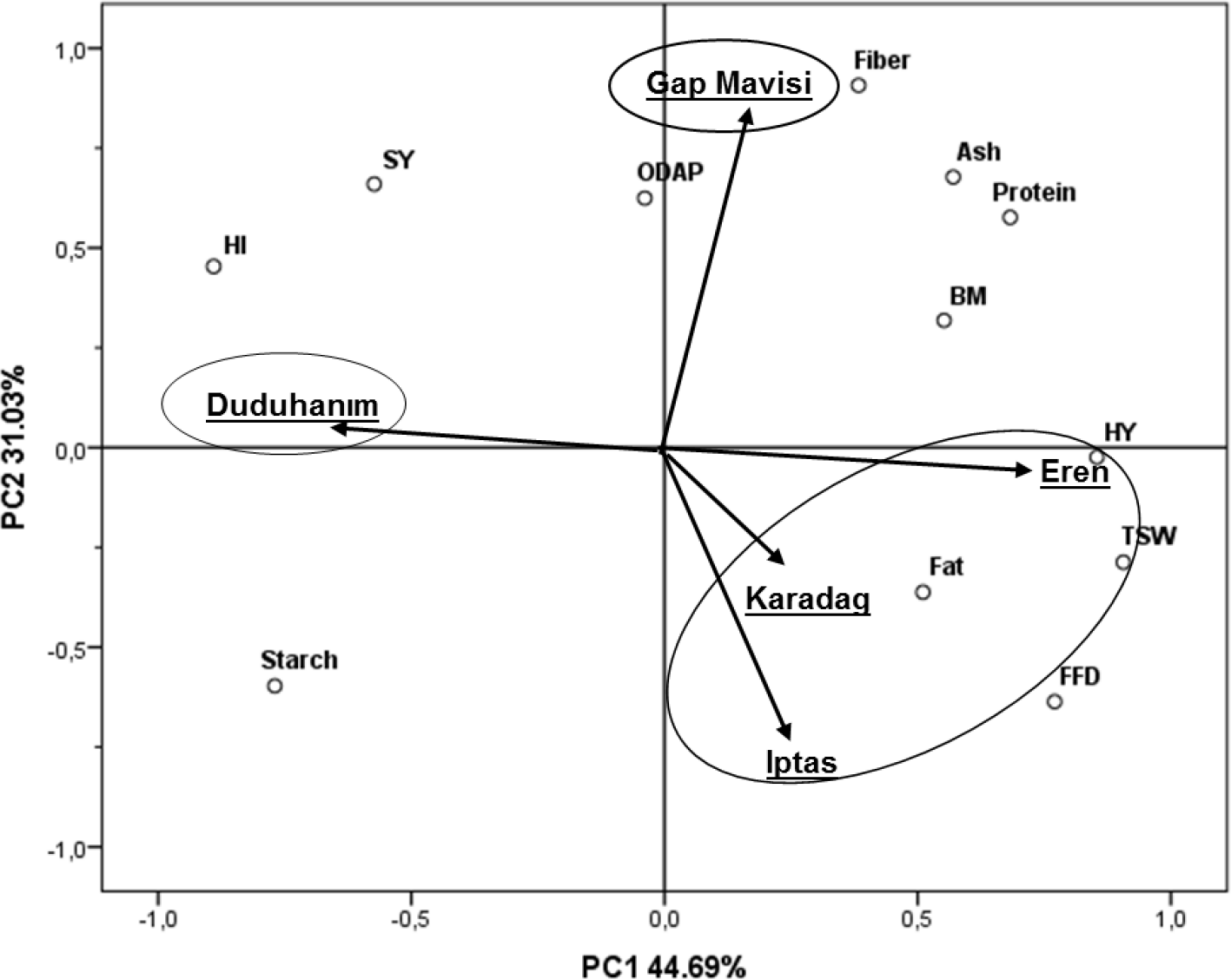
Principal component analysis expressing the variation of Türkish grass pea varieties based on the investigated traits.

Principal Component Analysis (PCA) and the biplot graph were created with the first two components, representing 75.72% of the meaningful total variance (**Figure 4**). The first component (PC1) explained 44.69% of the total variance and was loaded mainly by harvest index, hay yield, and thousand-seed weight, while the second component (PC2) explained 31.03% and was loaded by β-ODAP and fiber mainly. Grass pea varieties used in this study are distributed in 3 groups depending on the examined characteristics. Out of the three principal component groups, one included Duduhanım, the other Gap Mavisi, and the latter included Eren, Iptas and Karadag varieties. Accordingly, Duduhanım differed from the others by showing a high harvest index, seed yield, starch content, earliness, and low thousand-seed weight. Gap Mavisi attracted attention, especially with its high fiber, β-ODAP, ash contents, high seed yield, and protein contents. Eren, Karadag, and Iptas seemed similar and differed from the others mainly by their high-fat contents, hay yield, thousand-seed weight, high thousand-seed weight, low seed yield and harvest index.

## 4 Discussion

Grass pea is an annual legume known for its drought tolerance, yield, high adaptability, and nutritional value. Despite all these superior qualities, its consumption and cultivation are restricted due to its B-ODAP content. In this context, grass pea breeding efforts worldwide have focused on developing varieties with high yield and nutritional value, particularly those with low ODAP content (Hanbury et al., 2000; Basaran et al., 2016). However, paradoxically, in grass pea breeding studies in Türkiye, ODAP is not included as a standard characteristic in registration processes and therefore is not considered when registering new grass pea varieties. The main determinants in grass pea registration procedure are yield, protein contents, and phenological characteristics (such as earliness) in Türkiye. Consequently, compared to world varieties, the slightly higher β-ODAP concentration (3.99 mg g^-1^) and the consistent seed yield (3.11 t ha^-1^) and protein content (28.52%) in Turkish grass pea varieties can be attributed to this procedure.

Previously, highly variable data are reported in terms of seed yield among ecologies and genotypes in grass pea worldwide. Seed yield varied from 0.42 to 3.86 t ha^-1^ in Italy (Piergiovanni et al., 2011); from 1.32 to 1.8 t ha^-1^ in India (Campbell, 1997) and, up to 5.00 t ha^-1^ under very favourable conditions in Ethiopia. Ksiezak and Bojarszczuk (2020) reported the two-year mean seed yield of two grass pea varieties (Derek and Krab) under Polish conditions as 2.87 and 2.93 t ha^-1^, respectively. The seed yield of grass peas fluctuated between 1.14 and 3.09 t ha^-1^ in experiments conducted throughout Türkiye (Karadag and Yavuz, 2010; Sayar and Han, 2015; Basaran et al., 2016; Tenikecier, 2020). These studies were conducted under different ecological conditions and with different genotypes. Therefore, they demonstrate that there is still potential for improvement in grass pea seed yield in Türkiye. Furthermore, they highlight the importance of genotype-ecological compatibility and, consequently, variety development.

β-ODAP is significantly affected by genetics and environment (Grela et al., 2010; Mekonen et al., 2024) and varies greatly across genotypes from ~0.2 to 25.9 mg g^-1^ (Kumar et al., 2011). The β-ODAP content of >50 Türkiye grass pea cultivars, including landraces or varieties has been recorded in between 1.00 and 8.7 mg g^-1^ (Basaran et al., 2013, Basaran et al., 2016; Onar et al., 2014). Edaphic and climatic conditions have significant importance in raising or dropping the concentration of β ODAP in the grass pea. The β-ODAP content of the Australian variety Ceora is determined to be 0.4-0.9 mg g^-1^ in Australia (Siddique et al., 2006), while it is determined to be 3.52 mg g^-1^ in Türkish conditions (Arslan et al., 2017), which is a striking example of the environmental effect. It has been established that drought, excessive iron, cadmium, or zinc deficiency in the soil also cause increased β-ODAP content in grass pea (Liu et al., 2017). This shows that ecological factors play an important role in increasing the β-ODAP contents of the varieties. Stressors like high altitude (1300 m), high evaporation, and low precipitation also affected β-ODAP in current study. Quite low total precipitation was noted during the vegetation period (April-July) in 2022 (155.6 mm) and 2023 (204.4 mm). Therefore, the difference in β-ODAP between years may also be related to drought. Compared to 2022, total precipitation increased by 31% and β-ODAP decreased by 43% in 2023. From a different perspective, these results also indicate that the considered varieties may have much lower β-ODAP under appropriate growing conditions. Over the varieties, biomass yield, seed yield, hay yield, thousand-seed weight, protein, and fat contents increased in parallel with the increase in precipitation in the second year but harvest index, thousand-seed weight, and fiber decreased as expected. Ash was found similar in both years, which show higher stability of varieties for ash contents. Choudhury et al. (2022) reported the nutritional values ​​of several grass pea genotypes as follows: crude protein: 26.24% – 29.70%, crude fat: 1.19% – 2.34%, total carbohydrates: 50.85% – 55.34%, crude fiber: 6.57% – 8.67%, and ash content on a dry weight basis: 2.68% – 3.92%. Hanbury et al. (2000) reviewed the data of many studies and determined the average protein, ash, starch, fat, and fiber content of grass pea as 29.4%, 2.6%, 41.2%, 1.6%, and 8% in the same order. The nutritional values ​​determined in Türkish grass pea varieties are consistent with the results reported in previous studies.

The nutritional value of grass pea is determined by their biologically available nutrient content and the effects of anti-nutrients. Enneking (2011), based on the results of numerous studies, reports that increased consumption of grass pea seeds provides energy, protein, minerals, lipids, and vitamins for survival and maintenance, but over time it can lead to deficiencies, particularly in lipids, sulfur-containing amino acids, vitamins C and B12, and also, depending on food preparation methods, minerals and water-soluble vitamins. The author also reported that malnutrition is not necessary for the development of neurotoxicity, but the combined effect of low-sulfur amino acids and various anti-nutrient factors in grass pea can lead to the emergence of neurotoxicity. These negative effects can be mitigated with a balanced diet that includes cereals (Lambein and Kuo, 2004). These data emphasize the importance and necessity of consuming grass pea with foods rich in vitamins, minerals, and antioxidants for a balanced diet, especially in the long term.

Based on two-year averages, the Türkish origin varieties examined exhibited significant differences in flowering, grain yield, and crude protein content. B-ODAP content also varied, although not significantly. The most recently registered Duduhanım variety stands out among other varieties for its earliness, high harvest index, grain yield, and low thousand-seed weight. It has an average value among the varieties for crude protein and B-ODAP content. Early-maturing and high-yielding varieties are extremely important, especially under challenging growing conditions and drought stress. These varieties can enhance food security in drought-prone or water-scarce regions by both allowing escape from terminal drought, reducing production risks, and enabling the cultivation of a second crop. Shubha et al. (2025) investigated the morpho-agronomic characteristics of seventeen grass pea lines for suitability to dual-purpose (leaf and seed) and determined that seed-type genotypes flowered an average of one week earlier than leaf-type genotypes. Furthermore, the same authors found strong negative correlation between flowering time and seed yield (*r*=−0.750). These results support our findings. These results support our findings and explain why high-yielding varieties are also early-flowering. In this context, it is noteworthy that the Duduhanım variety, registered as a seed type, is earlier than other varieties and also among the most productive varieties. Therefore, early-flowering seed-type genotypes may be more advantageous and successful in arid regions.

In conclusion, grass pea varieties registered in Türkiye appear to be globally competitive in terms of yield and quality traits. Therefore, they could represent an important resource for global grass pea breeding efforts. However, the β-ODAP contents of Türkish origin varieties are slightly higher compared to varieties grown in other parts of the world. This can largely be attributed to climatic conditions and the pleiotropic effects of some genes. However, current β-ODAP values are not at the desired level. Therefore, in addition to improving yield, it is recommended that Türkiye focus on breeding varieties with low or zero β-ODAP content. Furthermore, developing specialized agronomic techniques for different geographic regions to reduce β-ODAP content and conducting studies to increase the yield potential of low-toxicity genotypes should be the priority goals of grass pea breeding in Türkiye.

## Data Availability

The raw data supporting the conclusions of this article will be made available by the authors, without undue reservation.
